# Step‐by‐step: A traction‐based fenestration method for vasoepididymostomy

**DOI:** 10.1002/bco2.70088

**Published:** 2025-09-14

**Authors:** Kosuke Kojo, Masahiro Uchida, Kazumitsu Yamasaki, Jaejeong Kim, Ayumi Nakazono, Daisuke Numahata, Takazo Tanaka, Hiroyuki Nishiyama, Tatsuya Takayama, Teruaki Iwamoto

**Affiliations:** ^1^ Department of Urology, Institute of Medicine University of Tsukuba Tsukuba Ibaraki Japan; ^2^ Center for Human Reproduction International University of Health and Welfare Hospital Nasushiobara Tochigi Japan; ^3^ Department of Urology Tokyo Medical University Ibaraki Medical Center Ami‐machi Ibaraki Japan; ^4^ Department of Urology Tsukuba Gakuen Hospital Tsukuba Ibaraki Japan; ^5^ Department of Medical Education, Institute of Medicine University of Tsukuba Tsukuba Ibaraki Japan; ^6^ Department of Urology St. Marianna University School of Medicine Kawasaki Kanagawa Japan

**Keywords:** LIVE, longitudinal intussusception vasoepididymostomy, male infertility, obstructive azoospermia, seminal tract re‐anastomosis

1

In this article, we present a practical technical tip for fenestrating the epididymal tubule during vasoepididymostomy (V‐E)—a step that, to date, has rarely been described visually. Using simple illustrations, we aim to provide a clear visual guide for this crucial part of the procedure. V‐E, a type of seminal‐tract re‐anastomosis for obstructive azoospermia, is regarded as one of the most technically demanding forms of male infertility microsurgery.^1^ The “intussusception method” (also known as the “invagination method”), in which the fenestrated epididymal tubule is pulled into the lumen of the vas deferens for an end‐to‐side anastomosis, is a widely adopted approach. Notably, “longitudinal intussusception vasoepididymostomy (LIVE)”—which involves placing two double‐armed needles longitudinally in the outer wall of the epididymal tubule, then incising the space between them—has been reported to be both simpler and more effective than other V‐E techniques. We also actively employ the LIVE method in our practice. Chan, one of the developers of LIVE, described using a 15° ophthalmic knife to make a longitudinal incision in the outer wall of the epididymal tubule during fenestration.^1^ However, we found it challenging to achieve a clean fenestration in a single pass, as the force applied by the microblade tip does not efficiently transmit to the soft outer wall. We suspect that, since Chan's original report, many surgeons have independently adopted minor modifications to overcome this challenge, but to our knowledge, such techniques have not been formally documented—likely due to their seemingly trivial nature.

Our technical tip for fenestration is illustrated in Figure 1. The only difference from Chan's method is that, instead of simply making an incision with a microblade, we use a needle to apply traction to the epididymal tubule wall while excising it with ophthalmic scissors. Specifically, after exposing the epididymal tubule from the tunic at the cauda (the portion closest to the vas deferens) and selecting the thickest and straightest segment, two double‐armed 10–0 nylon sutures are placed longitudinally, parallel to the axis of the tubule and as far apart as possible within the diameter of the tubule (approximately 300 μm). Our practical technical tip is summarized in two steps:

Step 1. A third 10–0 nylon suture is inserted between the two needles, at a depth shallower than the tubule's diameter, and gentle traction is applied. This manoeuvre lifts the tubule into a tent‐like configuration.
Step 2. Using ophthalmic scissors, the outer wall is partially excised by gliding the blades along the two needles of the sutures. The size of the fenestration can then be freely adjusted by modulating the tension on the traction suture.

**FIGURE 1:**
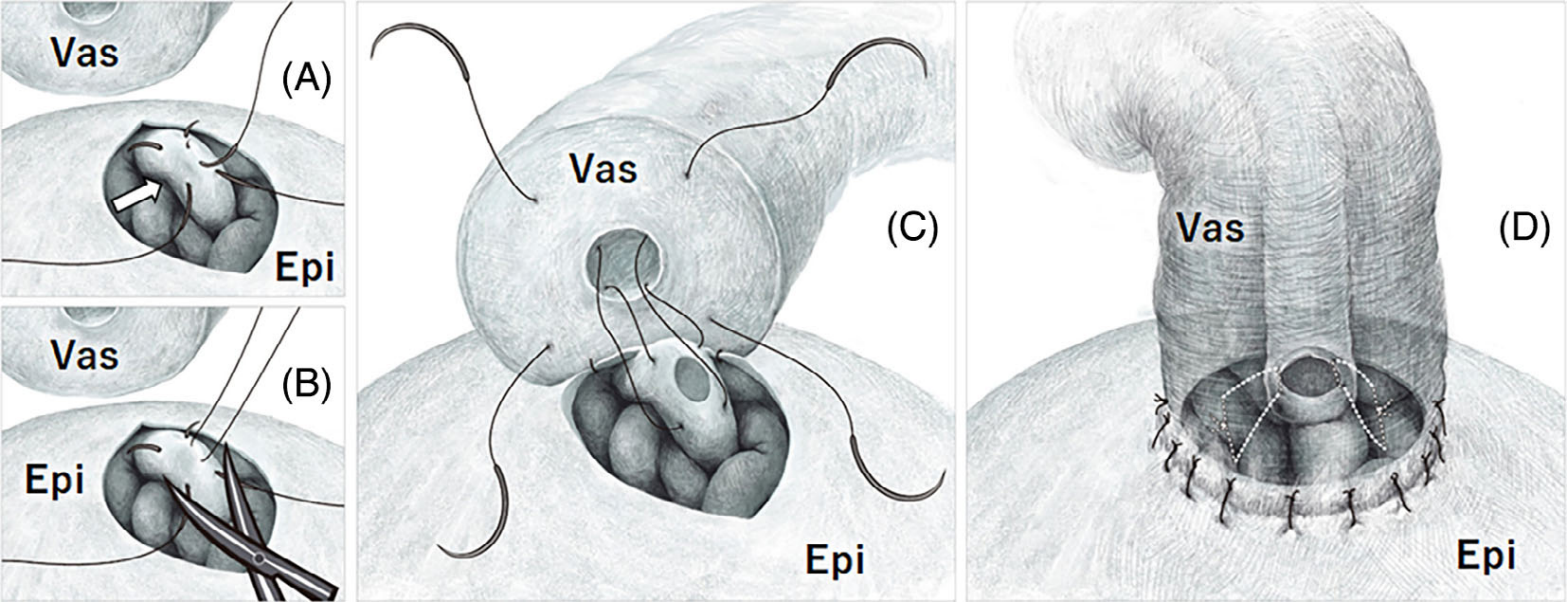
(a) **Step 1**. Two double‐armed sutures are placed through the exposed epididymal tubule (arrow), with a separate traction suture passed between them. (b) **Step 2**. While gently lifting the traction suture, ophthalmic scissors are used to excise a portion of the outer wall of the epididymal tubule. (c) The double‐armed sutures are advanced through the vas deferens mucosa. (d) Final appearance after completion of the anastomosis. Vas, Vas deferens; Epi, Epididymis.

After fenestration, the fluid leaking from the site is placed on a slide and examined immediately to confirm the presence of sufficient sperm. If no sperm are detected, a new fenestration is made slightly closer to the caput (the testicular end) of the epididymis, and the process is repeated. Fenestration sites not used for anastomosis are closed using absorbable suture and the tunica of the epididymis. Once a successful fenestration is achieved, we proceed with the standard LIVE technique: using the initially placed needles, we suture the mucosa of the vas deferens from inside to outside at four points, tying each suture to pull the epididymal tubule into the lumen of the vas deferens. Finally, we complete the anastomosis by suturing the tunica of the epididymis to the outer layer of the vas deferens with 9–0 nylon. A brief narrated video demonstrating Steps 1 and 2 and their integration into the standard LIVE workflow accompanies this article (Video 1).

Since 2015, our team has adopted this approach across multiple institutions, but some limitations of this visual technical tip should be noted. First, we did not directly compare clinical outcomes of this modification to those achieved with Chan's original LIVE method. Second, we have not quantitatively evaluated the extent to which this technique reduces the technical stress experienced by surgeons when fenestrating the epididymal tubule. As Supporting Information to a previously published case report, we have already disclosed a retrospective summary of the outcomes of 19 LIVE procedures performed by four surgeons in our team between 2015 and 2019.^2^ In these cases, which were followed for at least one year postoperatively, there were no perioperative complications exceeding Clavien‐Dindo grade I. Of the 19 patients, sperm were detected postoperatively in 14, and 7 partners achieved pregnancy (3 by natural conception and four by assisted reproductive technology). In recent years, there has been a trend in Japan toward favouring testicular sperm extraction, which is considered more reliable, over seminal‐tract re‐anastomosis for the treatment of obstructive azoospermia. This has led to a low nationwide case volume, making definitive statistical comparisons difficult. Nonetheless, our observed success rate of 73.7% (14/19) is at least comparable to, if not higher than, the 42–61% reported in nationwide surveys on V‐E in Japan.^3^, ^4^ Moving forward, it will be important to collaborate with other surgeons who adopt this technique to systematically evaluate the learning curve, operative time and cost‐effectiveness. We hope that this visual technical tip will expand the available options for both surgeons and patients and contribute to education and improved shared decision‐making in male infertility surgery.

## AUTHOR CONTRIBUTIONS

KK and TI conceptualized the study. KK, MU, KY, AN, DN, TT and TI conducted the investigation. KK wrote the original draft of the manuscript. KK, KY and JK prepared the visualizations. HN, TT and TI supervised the project.

## CONFLICT OF INTEREST STATEMENT

The authors declare no conflict of interest.

## Supporting information


**Video S1.** **Supporting Information**.
00:00–00:19 Exposure of the target epididymal tubule.
00:19–00:39 Placement of first and second sutures.
00:39–00:51 **Step 1.** Placement of a third suture.
00:51–00:56 **Step 2.** Fenestration.
00:56–01:14 Sperm check.
01:14–01:40 The process of anastomosis.
01:40–01:49 Final appearance after completion of the anastomosis.
